# Using telehealth clinical case vignettes to enhance clinical confidence and competence in veterinary students

**DOI:** 10.3389/fvets.2022.1075752

**Published:** 2023-01-16

**Authors:** Brian V. Lubbers, Virginia R. Fajt, Lori M. Teller, Michael D. Apley, Jacqueline Stillisano

**Affiliations:** ^1^Department of Clinical Sciences, College of Veterinary Medicine, Kansas State University, Manhattan, KS, United States; ^2^Veterinary Physiology and Pharmacology, School of Veterinary Medicine, Texas A&M University, College Station, TX, United States; ^3^Veterinary Small Animal Clinical Sciences, School of Veterinary Medicine, Texas A&M University, College Station, TX, United States; ^4^School of Education and Human Development, Texas A&M University, College Station, TX, United States

**Keywords:** telehealth, case discussions, day-one competencies, curricula, remote instruction and teaching

## Abstract

Veterinarians contribute substantially to the health of their patients and enhance the communities in which they live. Delivery of veterinary curricula continues to evolve to ensure that veterinary graduates are prepared to meet their professional obligations on Day One of their careers. In this study, veterinary practitioners were recruited to deliver telehealth case rounds to veterinary students at Kansas State University and Texas A&M University. Case discussions were hosted virtually once per month in the 2020–2021 and 2021–2022 academic years for a total of 16 sessions. Each presenting practitioner was instructed to develop a brief presentation for a case routinely seen in their practice, and to discuss important clinical decision points in diagnosis, treatment and management. Cases could also highlight important ethical or communication issues encountered in veterinary medicine. The overall goals of this project were to increase the quantity and diversity of clinical cases to which veterinary students were exposed during their professional training and to evaluate the feasibility and acceptability of telehealth technology as a teaching strategy. Student participants were surveyed to determine the effectiveness of telehealth sessions in increasing overall confidence and competence in case management, and veterinary presenters were surveyed to determine motivations for participating in the project and perceived value of the telehealth sessions. More than 95% of students indicated that participation in telehealth sessions increased their clinical confidence and competence. Presenting practitioners unanimously indicated that they would participate in similar instruction in the future. Recommendations are provided to improve the educational experience for future adopters of telehealth teaching sessions.

## Introduction

Veterinarians protect animal health and welfare and promote public health. They ensure that our food is safe, support rural and animal production economies, and enhance the communities that they live in. To prepare veterinarians to fulfill these professional obligations, veterinary education focuses on a set of core competencies to make new graduates practice-ready (1, 2). To address the need for practice-ready veterinary graduates, veterinary curricula, as a whole, are evolving from primarily subject-based to a competency-based teaching model (3). Recognized learning outcomes include clinical reasoning and decision-making, individual animal care, animal population management, public health issue awareness, effective communication skills, ability to establish or foster a collaborative environment (including leadership skills), attitude of professionalism, financial and practice management, and scholarship (1). Previous reports suggest that the transition from university study to veterinary practice is difficult due to lack of experience with technical skills, management of primary cases and client communication (4). These outcomes are difficult to achieve in a traditional, content-based curriculum, and a recent survey of veterinary administrators demonstrated that minimal progress has been made in breaking the cycle of memorize, regurgitate, forget (5).

Although case-based learning has been touted as an effective teaching strategy, there is little evidence to support these claims (6). Studies do show, however, that both students and teachers enjoy case-based learning and that this strategy enhances student engagement in the classroom and overall confidence (7, 8). Therefore, case-based learning may augment the development of clinical competence, confidence and skills to be a practice-ready veterinary graduate.

**Figure 1:**
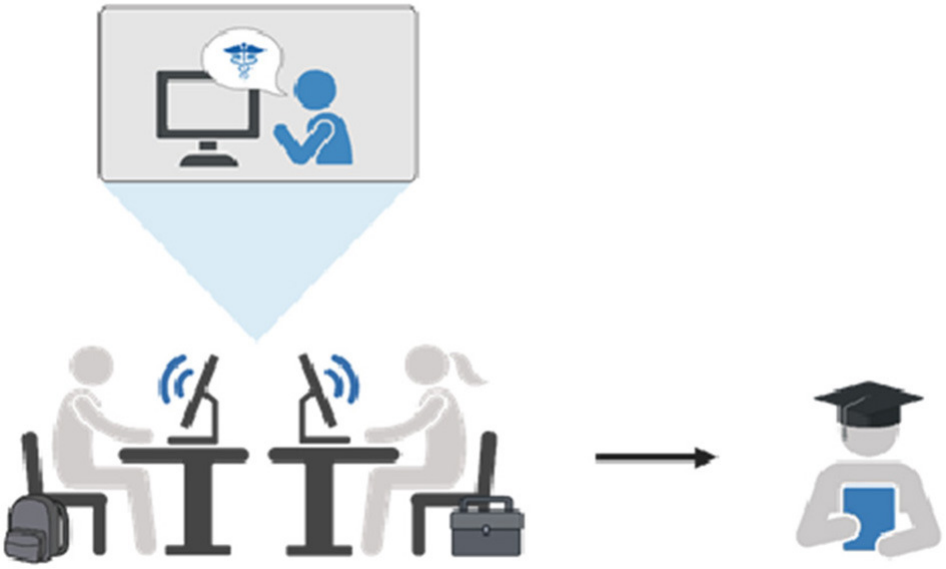
Information flow in telehealth case vignettes.

Currently, the majority of clinical decision-making experience comes to veterinary students during their clinical years, primarily the final year in many veterinary education programs. The caseload encountered during the clinical year(s) may not be reflective of the type of cases seen in primary care practice. Our objective was to provide students with additional opportunities (earlier in the curriculum and more frequent exposure) to work through cases commonly encountered in private practice settings, including frank discussions of the financial and communication challenges encountered in primary care settings. To incorporate additional first-opinion, primary care cases into veterinary training, we developed an approach that reverses the Project Extension for Community Healthcare Outcomes (ECHO) model of care delivery (9). Project ECHO was initiated to increase the availability of expertise in rural and resource-limited human healthcare settings (10). ECHO consists of a network with a central node that is advanced expertise, e.g., an academic institution or specialty medical center, and other nodes being community care providers, with the network connecting expertise to primary care health professionals to manage complex medical conditions (Figure 1).

Although the ECHO model supports collective learning, information generally moves from the central hub outward. The approach reported here, also intended to support collective learning, was established to draw information into the central hub from practice settings that routinely manage the clinical cases that veterinary graduates will experience on day one of their professional career. The overall goal of this project was to increase the quantity and diversity of clinical cases to which veterinary students were exposed during their professional training. A secondary goal of this project was to evaluate the feasibility and acceptability of telehealth technology to enhance student exposure to mixed-animal, primary care cases.

## Materials and methods

This project was reviewed by the Institutional Review Boards at Kansas State University and Texas A&M University and met the criteria for exempt research (11).

Veterinary practitioners in first-opinion, small and large animal-oriented practices were recruited from the investigators' professional networks. Each practitioner was asked to present a case representative of typical cases in their practice (>12 cases/year). Cases that highlighted “soft” skills such as client communication, professional ethics, or the economics of veterinary practice were encouraged. It was emphasized to participating practitioners that each case did not have to have a final diagnosis/clearly established etiology to meet the requirements of the project.

Practitioners were instructed to follow the format in Table 1 for each session. They were also provided an optional PowerPoint template for developing the case presentation.

**Table 1:** Structured format for telehealth case vignettes presented by private practitioners to preclinical veterinary students.

**Item**	**Time (min)**	**Assigned to**
Host welcome, purpose statement, ground rules	1	Host facilitator
Case history and clinical presentation	2–3	Presenting practitioner
Questions, discussion and suggested diagnostic plan	2–5	Students
Actual diagnostic plan and results	2	Presenting practitioner
Questions, discussion and suggested treatment plan	2–5	Students
Actual treatment plan and case outcome	2	Presenting practitioner
Session wrap-up/final comments	3	Host facilitator
**Total time**	**14–21 min**	

Telehealth sessions were co-hosted *via* Zoom by project collaborators at Kansas State University and Texas A&M with students from both schools attending each session. Four telehealth sessions were held each fall and spring semester during the 2020–2021 and 2021–2022 academic years, for a total of 16 telehealth sessions. Students at Texas A&M University were invited *via* email before each session and a Zoom link was provided for participation. For all but one session, Texas A&M University students attended individually *via* Zoom. Students at Kansas State University were also invited *via* email before each session and a Zoom link was provided for participation. However, sessions were held in conjunction with monthly meetings of the KSU Food Animal Veterinary Certificate program; therefore, the majority of Kansas State students met in person (as a group) with the veterinary practitioner on Zoom. At the end of each session, a link to a questionnaire was shared with all students for feedback (Appendix 1 lists the actual survey questions).

A written summary of each case was developed by the project team following each session. Summaries were approximately 2–3 pages each and highlighted pertinent aspects of the case and provided additional references. The summary materials were posted to a password protected website (made available to attendees or by permission of the research team) along with video recordings of the case presentation.

At the conclusion of all sessions, presenters were contacted *via* email to participate in in-depth, semi-structured interviews conducted by one of the authors (JS) *via* Zoom. Lasting about 25 min each, the interviews were audio recorded and transcribed for later analysis. The protocol was designed to collect qualitative data from participating veterinary practitioners regarding (a) motivating factors for participating in the Telehealth Vignette Project, (b) aspects of the project that were identified by the presenting practitioners as most and least valuable for themselves, and (c) opinions regarding the value of the experience for the veterinary students with whom they interacted. Although the questions on the interview protocol established the structure and focus of each interview, participants were encouraged to reflect on their responses and expand their expositions.

### Data analyses

Responses to the student surveys completed after each session were summarized across all the sessions due to the low number of completed surveys. Descriptive analysis (percent of students reporting levels of agreement/disagreement) of the survey responses was conducted. Free text comments on the student surveys were reviewed to improve future sessions (data not shown).

Qualitative data from the veterinary practitioners were analyzed using a general inductive approach, guided by specific research objectives (12). Detailed and repeated readings and interpretations of the raw data generated multiple codes, which were sorted into categories or families (13) and used to create a framework of the most significant themes related to the evaluation questions.

## Results

A total of 144 completed student evaluations were received for the 16 telehealth sessions. As data were collected anonymously, it was not possible to determine how many evaluations were submitted by the same students or if different students responded after each of the sessions. Seven of the 16 practitioners completed interviews with the evaluation team.

Student survey responses and practitioner interview responses are described below in the context of 3 areas: the concept of case delivery as a model for teaching, session format and overall impact or value.

### Model for teaching

Nearly all student responses (99%) indicated “agree” or “strongly agree”, with more than 90% of students “strongly agreeing”, that presenting practitioners were knowledgeable regarding the cases and that practitioners provided thorough explanations of the materials and answers to student questions.

Practitioners were asked what aspects of the telehealth sessions were valuable to the students. Overall, presenting practitioners rated engagement (as an indicator of the importance of a learning experience) as “moderate”. Respondents hypothesized that the virtual environment and larger group settings may have contributed somewhat to reduced engagement. However, many respondents were more positive regarding the impact that participating in these sessions would have had on their early career experience.

- “I think that the cases really helped to drive concepts home and make things more applicable and in real world, so I think it would have been very valuable.”
- “I think it would have been very, very valuable.... It would have maybe made me more confident in my 1^st^ year or two out of school... because that's the thing—we have all the knowledge, and the university does a great job of preparing us. It's, you know, just really difficult to bridge that gap between a specialty hospital and a frontline practitioner, out in rural America.”

### Session format

While nearly all student responses (98%) indicated agreement or strong agreement with the appropriateness of the allotted time (20–30 min) for telehealth sessions, the overall rate of strongly agree was 75%. Likewise, 90% of students answered in the affirmative that materials supported interactive learning, although only 57% of students strongly agreed.

During the presenter interviews, most practitioners agreed that the time period allotted for the sessions was “about right” or “adequate”. However, a few presenters felt that the sessions were a little short to present the entire case.

Practitioners were also questioned about their level of comfort with presenting cases to students in a virtual environment both before and after participation in the telehealth sessions. Although one practitioner had expressed a “lack of experience” with virtual teaching, most practitioners expressed moderate to high levels of comfort prior to presenting and many expressed that they were more confident after participating. One practitioner was very pleased with the experience:

- “I felt a lot more comfortable after....Now that I have my PowerPoint slides built, I can just think of a million different cases that I could go through....After challenging myself to do it, now I would be a lot more confident in doing another one...or even being able to call Dr. Lubbers and say, “Hey, I have a really neat case that I just went through. I could present it at any time.”
- Presenting veterinarians were also asked about the extent to which participation in the sessions had been an inconvenience or too much of a commitment. Most presenting veterinarians agreed that development of the case materials and the actual presentation was not excessive and that the benefits of participating outweighed the time commitment.

### Overall impact or value

As for the overall impact and value of participating in the telehealth sessions, more than 95% of students indicated that participation increased their confidence and competence. Approximately half of the student responses indicated that they did NOT need additional instruction regarding the case materials.

Many of the presenting practitioners identified **professional self-review** as a primary value to themselves for project participation. Practitioners expressed the following:

- “I think it was a good opportunity to review—kind of review some of the cases that I've had over the years and think about how I approach cases, and kind of make sure, have I gotten into ruts? Am I jumping to conclusions or am I going through the correct or the recommended workup whenever things come in?”
- “You know, sometimes we go through the motions, and we do a lot of things right, but we don't think about why we do them....So it was kind of a fun challenge to go through and just make sure....I just wanted to make sure that I was presenting the best opportunity I could for the students, and that was a fun challenge for me.”

When asked if they would be willing to present again at a future date, participating veterinarians unanimously agreed that they would. Participants expressed enthusiasm for the opportunity to teach and interact with students as reasons to present additional cases in this format. Other participants believed that these sessions could serve as another point of contact with students for externships or future employment opportunities. Although a few practitioners reported follow-up requests for externships or an increase in social media followers after their presentation, most practitioners indicated that, at the time of their interview, they had not had any follow-up interactions with veterinary students directly related to the telehealth sessions.

## Discussion

Case-based discussions have been used for a long time in veterinary education (14). They have been shown to be enjoyed by students and educators in the health professions, although there are equivocal data about their effectiveness (6). Remote delivery of veterinary education has been described, e.g., by Sim et al. (15), but we did not find any published reports of approaches similar to ours of having private veterinary practitioners deliver primary-care case discussions to veterinary students in a remote manner.

In our study, student and practitioner responses were generally positive regarding the use of virtual case presentations as a model for teaching and the session format. Participants indicated that the telehealth sessions were valuable in increasing student exposure and confidence in the types of cases seen in primary-care veterinary practices. This suggests that the benefits to students, faculty, practitioners and the veterinary profession as a whole, outweigh the effort needed to conduct virtual case presentations. We identified several areas for improvement to increase the value of this type of student-practitioner interaction, and we make recommendations for implementing a similar program (Table 2).

**Table 2:** Activity recommendations for teaching faculty, veterinary practitioners and students during planning and conduct phases of telehealth case discussions using a remote platform.

**Team member**	**Planning**	**Before sessions**	**During sessions**	**After sessions**
Faculty leads	Define learning objectives	Invite students (if sessions are not part of a scheduled course or student activity)	Moderate engagement between practitioners and students	Request feedback from practitioners and students
	Create session template	Review presentation materials, when requested	Identify learning opportunities related to the primary care nature of the cases or the practice setting	Reflect on student/practitioner interactions to identify successes and opportunities for improvement
	Identify time for sessions (in a course, student club, or other activity)	Schedule remote meeting/provide meeting invitation to all participants		
	Develop practitioner network			
	Schedule practitioners			
Veterinary practitioners	Consider case possibilities and develop case materials	Ensure equipment is available and adequate; block off time for the session	Invite student participation by pausing long enough for student responses	Provide feedback to faculty
Students		Commit to attending and engaging	Participate in an engaged manner	Contact practitioners for additional learning opportunities

First, communication with both students and practitioners about expectations for the sessions is critical. For example, one of the student critiques of the experience was the case selection by practitioners. While these sessions were intended for students interested in rural, mixed-animal practice, and the project team expected cases to include cattle, horses, dogs, and cats, student participants at Kansas State University were members of the Food Animal Veterinary Certificate program. Therefore, some students expressed an interest in having a greater focus on production animal cases. Additionally, practitioners expressed some disappointment with the lack of student engagement and follow-up regarding externship and employment opportunities. While the practitioners hypothesized that the lack of engagement may have been related to the virtual environment and student group size, our hypothesis is that it may be related to the student audience: for most sessions, based on informal assessment by the faculty members in the room or online, it was composed primarily of 1^st^ and 2^nd^ year students (pre-clinical). Students in the 1^st^ and 2^nd^ year of a veterinary curriculum may have limited exposure to the foundational coursework necessary to develop a differential disease list, diagnostic workup or treatment plan. In hindsight, it may have benefitted both students and presenters to set more realistic expectations of the students' experience and knowledge-base and their immediate need (low) for externships or post-graduate employment.

Another aspect of the sessions that we would eliminate for future iterations is the use of written case materials. Although we spent time writing and editing case descriptions and general discussion about diagnosis, treatment, and management related to the cases presented, these materials added very little value to the overall student experience and could easily be omitted in future programs that use virtual case presentations, especially given the number of educational resources available to students. One major issue with integrating these materials into the student experience during this project was the delay between the case presentation and completion of the written summary, since we were not always aware of what case the practitioners were going to present prior to the session. Although these written materials could be used to supplement future use of these case discussions, we believe that the most valuable aspect of the case presentations and interactions between students and practitioners was not related to knowledge of the medical condition but rather in the discussions about prioritizing clients' financial constraints, managing communications with clients and client expectations, and understanding how veterinarians in private practice think through case management.

This project was conceived by the investigators prior to the COVID-19 pandemic but was conducted through it. The initial project proposal even included the possibility that we would need to send microphones or other equipment to practitioners to facilitate the sessions. The ultimate impact on student learning during the pandemic is still unknown; however, this project provides some insight into virtual educational sessions. When students are attending all their classes virtually, it was not possible to have them in a classroom with a faculty member. This likely led to the reduced engagement and student participation, such as asking questions or responding when prompted for questions about the case. On the other hand, the impact of COVID was positive in terms of working with veterinarians in a virtual teaching environment. Practitioner survey responses indicate that this presented almost no issue at all, likely due to familiarity with virtual meeting platforms by the time sessions were initiated in fall 2020. Anecdotally, internet connectivity and sound and video quality were more than adequate to conduct the telehealth sessions. If these issues are of concern, adopters of telehealth teaching strategies could use pre-presentation test runs and provision of affordable webcams to resolve the situation. Familiarity with virtual platforms, managing remote meetings, and toggling between listening and talking are common now, even among those living in rural areas, which bodes well for implementing other remote teaching models.

## Data availability statement

The datasets presented in this article are not readily available because to meet IRB criteria of exempt research, participation in surveys was voluntary and anonymous. Cumulative anonymous data are insufficient to replicate the study reported here. Requests to access the datasets should be directed to BL, blubbers@vet.k-state.edu.

## Ethics statement

The studies involving human participants were reviewed and approved by Kansas State University IRB and Texas A&M University IRB. Written informed consent for participation was not required for this study in accordance with the national legislation and the institutional requirements.

## Author contributions

BL designed the study, led the study, collected and analyzed data, and wrote the draft manuscript. VF designed the study, collected and analyzed data, and wrote the draft manuscript. LT and MA designed and conducted the study. JS designed the survey, conducted interviews, and wrote part of the manuscript. All authors contributed to manuscript revision, read and approved the submitted version.
