# Supplementary material for: Using telehealth clinical case vignettes to enhance clinical confidence and competence in veterinary students
Source: Front Vet Sci. 2023 Jan 16;9:1075752. doi: 10.3389/fvets.2022.1075752 (PMC9884685; doi:10.3389/fvets.2022.1075752)
Supplement: Supplementary file 1 [file Data_Sheet_1.docx]

**Appendix 1.** Student post-session evaluation questions. Participating students were asked to select their level of agreement with these statements using the following scale: Strongly Disagree, Disagree, Agree, Strongly Agree.

| 1. The presenter was knowledgeable about the case materials. |
| --- |
| 2. The presenter answered questions carefully and completely. |
| 3. The presenter provided a thorough explanation of the case materials. |
| 4. The presenter applied sound teaching practices during the sessions. |
| 5. Participants were encouraged to generate ideas and questions about the case materials. |
| 6. The time frame allotted for the sessions was appropriate. |
| 7. The materials supported interactive learning. |
| 8. The resources provided were useful to me in learning about the case materials. |
| 9. The resources enhanced my understanding of the case materials. |
| 10. Overall, I still need more instruction regarding the case materials. |
| 11. Overall, the instructions provided regarding the case materials increased my confidence in using them. |
| 12. Overall, the instructions provided regarding the case materials increased my competence in using them. |
